# Dysfunction in differential reward-punishment responsiveness in conduct disorder relates to severity of callous-unemotional traits but not irritability

**DOI:** 10.1017/S0033291721003500

**Published:** 2023-04

**Authors:** Ru Zhang, Joseph Aloi, Sahil Bajaj, Johannah Bashford-Largo, Jennie Lukoff, Amanda Schwartz, Jamie Elowsky, Matthew Dobbertin, Karina S. Blair, R. James R. Blair

**Affiliations:** 1 Center for Neurobehavioral Research, Boys Town National Research Hospital, Boys Town, NE, USA; 2 Department of Psychiatry, Indiana University School of Medicine, Indianapolis, IN, USA

**Keywords:** Conduct disorder, callous-unemotional traits, irritability, reward responding, fMRI

## Abstract

**Background:**

Conduct disorder (CD) has been associated with dysfunction in reinforcement-based decision-making. Two forms of affective traits that reflect the components of CD severity are callous-unemotional (CU; reduced guilt/empathy) traits and irritability. The form of the reinforcement-based decision-making dysfunction with respect to CD and CU traits remains debated and has not been examined with respect to irritability in cases with CD. The goals of the current study were to determine the extent of dysfunction in differential (reward *v*. punishment) responsiveness in CD, and CU traits and irritability in participants with CD.

**Methods:**

The study involved 178 adolescents [typically developing (TD; *N* = 77) and cases with CD (*N* = 101)]. Participants were scanned with fMRI during a passive avoidance task that required participants to learn to respond to (i.e. approach) stimuli that engender reward and refrain from responding to (i.e. passively avoid) stimuli that engender punishment.

**Results:**

Adolescents with CD showed reduced differential reward-punishment responsiveness within the striatum relative to TD adolescents. CU traits, but not irritability, were associated with reduced differential reward-punishment responsiveness within the striatum, rostromedial, and lateral frontal cortices.

**Conclusions:**

The results suggest CD is associated with reduced differential reward-punishment responsiveness and the extent of this dysfunction in participants with CD is associated with the severity of CU traits but not irritability.

## Introduction

Conduct disorder (CD) is a serious behavioral and emotional disorder that emerges in childhood and adolescence. It is characterized as a repetitive and persistent pattern of behavior in which the basic rights of others and/or major age-approximate social norms are violated (APA, 2013). CD is associated with neurocognitive impairments in emotion recognition (Blair, Veroude, & Buitelaar, 2018; Fairchild et al., 2019), empathic accuracy (Martin-Key, Brown, & Fairchild, 2017), decision-making and reinforcement learning (Blair et al., 2018; Budhani & Blair, 2005; Fairchild et al., 2019). Dysfunctions in reinforcement-based decision-making are hypothesized to increase the risk of frustration-based reactive aggression and antisocial behavior more generally (Blair et al., 2018).

Core brain regions implicated in responding to reward and punishment include the striatum, ventromedial prefrontal cortex (vmPFC), posterior cingulate cortex (PCC), anterior insula cortex (AIC), anterior cingulate cortex (ACC), and inferior frontal gyrus (Clithero & Rangel, 2014; O'Doherty, Cockburn, & Pauli, 2017; Schoenbaum, Takahashi, Liu, & McDannald, 2011). These systems are involved in both reinforcement anticipation and the response to received reinforcement (Clithero & Rangel, 2014). A series of studies have indicated reduced reward responses in patients with CD relative to typically developing (TD) adolescents in regions including the striatum, vmPFC and PCC (e.g. Cohn et al., 2015; Finger et al., 2008; Hawes et al., 2020; White et al., 2013). Many of these studies have used tasks, such as the Monetary Incentive Delay (MID) task, where reward receipt is determined by instructed response (‘respond when the cue is present’; Cohn et al., 2015; Hawes et al., 2020; Rubia et al., 2009). While such tasks are useful for examining reward responsiveness, they do not allow the examination of a crucial functional significance of reward that the striatum is implicated in, i.e. its ability to change behavior via instrumental (and other forms of) learning (O'Doherty et al., 2017). Instrumental learning is of particular clinical interest with respect to CD and those at increased risk for antisocial behavior, given suggestions that deficits in instrumental learning increase the risk for antisocial behavior (Blair, Leibenluft, & Pine, 2014; O'Brien & Frick, 1996). Specifically, it has been suggested that deficits in instrumental learning mean that the individual is less likely to learn the ‘badness’, the negative value of antisocial actions (Blair et al., 2014). To learn in an instrumental learning task, the individual must be responsive to reward and punishment information and particularly differentiate the two. However, there remains considerable debate regarding whether the increased risk for antisocial behavior reflects reduced responsiveness to reinforcement information (Blair et al., 2014), increased responsiveness to reward such that the individual does not consider the potential *costs* of their actions (e.g. Buckholtz et al., 2010; Frick, Ray, Thornton, & Kahn, 2014) or a response set bias such that attention is increased to reward-related information but decreased to punishment-related information (Newman, 1998).

The diagnosis of CD is associated with affective traits, particularly callous-unemotional (CU) traits (Frick & Viding, 2009). CU traits reflect reduced guilt, empathy for others in distress and concern about one's own performance (Frick et al., 2014). Considerable work has examined the relationship between CU traits and threat processing (e.g. Aggensteiner et al., 2020; Fairchild, Van Goozen, Stollery, & Goodyer, 2008; Hodsoll, Lavie, & Viding, 2014; Marsh et al., 2011). Rather less work has examined the relationship between CU traits and reinforcement processing. Moreover, findings have been equivocal. Some studies have indicated no relationship between CU and reward responsiveness (Byrd, Hawes, Burke, Loeber, & Pardini, 2018; White et al., 2013). Others have reported that CU traits are negatively associated with reward responsiveness within vmPFC (Veroude et al., 2016) and the amygdala (Cohn et al., 2015; Schwenck et al., 2017). There have also been reports that CU traits are associated with increased striatal responsiveness – though only when others, not the self, receive reward (Schwenck et al., 2017).

A second important associated affective trait is irritability. Irritability is defined as an ‘increased propensity to exhibit anger relative to one's peers’ (Leibenluft, 2017, p.277) and a ‘relative dispositional tendency to respond with anger to blocked goal attainment, and includes both mood (trait) and behavioral (reactive state) dysregulation’ (Camacho, Karim, & Perlman, 2019; Fishburn et al., 2019, p.69; see also Stringaris et al., 2012; Wakschlag et al., 2018). Individuals with irritability show increased hostile attribution biases (Stoddard et al., 2016), a risk factor for conduct problems (Steinberg & Dodge, 1983). One recent study, using the MID task, has reported increased striatal responsiveness to reward as a function of the level of irritability in adolescents from intervention-seeking families in the community (Kryza-Lacombe et al., 2021). In addition, several studies have examined frustration in youth with severe mood dysregulation (SMD). One suggested typical responses to reward (Deveney et al., 2013). Moreover, they reported both increased and decreased responses within the dorsomedial frontal cortex (dmFC), dorsolateral prefrontal cortex (dlPFC), AIC/inferior frontal cortex (iFC) and striatum to negative feedback or information on the incorrectness of responses (Adleman et al., 2011; Deveney et al., 2013; Rich et al., 2011; Tseng et al., 2019).

The current study focuses on the association of CD, CU traits and irritability with differential response to the *receipt* of reward and punishment in the passive avoidance (PA; instrumental learning) task. Notably, the presence of both traits is relatively high in many individuals with CD and their severity is positively correlated even if the pathophysiology putatively underpinning both is markedly different (Blair, 2018; Brotman, Kircanski, Stringaris, Pine, & Leibenluft, 2017; Leibenluft, 2017). Surprisingly, although CU traits and irritability can be elevated in the same person, the research on them, for the most part, has developed in parallel (though see Blair, 2018; Wakschlag et al., 2018). In this study, we used a PA fMRI task with a relatively large sample (*N* = 178) of TD adolescents and adolescents with CD (CD: *N* = 101). We hypothesized that adolescents with CD would show reduced reward-punishment differential responsiveness within regions implicated in reinforcement processing (i.e. striatum, vmPFC, and ACC). Given the previous literature, predictions with respect to CU and irritability were less clear. As such, we simply hypothesized that if the level of CU traits and/or irritability was associated with the integrity of reinforcement processing in patients with CD, then the level of either or both traits would be associated with differential reward-punishment responsiveness within the striatum, vmPFC, and ACC.

## Methods

### Participants

We recruited 178 youths aged between 14 and 18 years of age from a residential care program and the surrounding community (age = 15.715 ± 1.805 years; IQ = 101.983 ± 11.411; 110 male). Youth recruited from the residential care program had been referred for behavioral and mental health problems. Participants from the community were recruited through flyers or social media. There were two groups of participants: participants with CD (*N* = 101; age = 15.874 ± 1.658 years; IQ = 100.792 ± 11.630; 65 male) and TD adolescents (*N* = 77; age = 15.507 ± 1.973 years; IQ = 103.546 ± 10.998; 45 male). The groups did not significantly differ in age, sex, or IQ (*p*'s > 0.10); see Table 1.

**Table 1.** Demographic and clinical variables

	Mean for CD	Mean for TD	*t* score	*p* value	Correlation with ARI for the CD group	Correlation with ICU for the CD group	Steiger's *Z*	*p* value
Age	15.874 ± 1.658	15.507 ± 1.973	1.345	0.180	0.072	0.158	−0.707	0.482
IQ	100.792 ± 11.630	103.546 ± 10.998	1.602	0.111	−0.295**	−0.121	−1.461	0.072
ARI	3.860 ± 3.263	0.829 ± 1.100	7.765	0.000	–	0.299**	–	–
ICU	24.490 ± 7.978	15.859 ± 6.856	7.330	0.000	0.299**	–	–	–
SDQ-CP	6.256 ± 2.242	0.339 ± 0.713	20.431	0.000	0.128	0.066	0.506	0.613
RPRS Total	16.985 ± 6.004	8.177 ± 2.608	10.644	0.000	0.081	0.074	0.057	0.955
RPRS Reactive	10.182 ± 3.486	4.839 ± 2.002	10.545	0.000	0.085	0.027	0.471	0.638
RPRS Proactive	6.821 ± 3.015	3.333 ± 0.861	8.847	0.000	0.064	0.114	−0.407	0.684
Conners (ADHD)	6.634 ± 6.813	0.351 ± 1.190	7.995	0.000	0.173	0.104	0.567	0.571
	Percent for CD	Percent for TD						
Male	56.7%	43.3%	0.802	0.424	−0.157	0.032	−1.544	0.123
MDD	38.8%	0%	4.063	0.000	0.224*	0.194	0.251	0.802
GAD	31.0%	0%	5.807	0.000	0.193	0.111	0.676	0.499
ADHD	80.0%	0%	17.030	0.000	0.153	0.055	0.801	0.423
CD	100%	0%	–	–	–	–	–	–
Antipsych-otic	13.6%	0%	2.660	0.009	0.031	0.037	−0.049	0.961
Stimulant	23.7%	0%	3.747	0.000	−0.074	−0.096	0.179	0.858
SSRI	16.9%	0%	3.035	0.003	−0.094	0.087	−1.474	0.140

MDD, major depressive disorder; GAD, generalized anxiety disorder; ADHD, attention deficit hyperactivity disorder; CD, conduct disorder; *p* = two-tailed significance level of the Steiger's *Z* calculation (i.e. whether there were significant differences in correlation strength between the ARI scores and ICU scores). *p < 0.05; **p < 0.01.

Clinical characterization was done through psychiatric interviews by licensed and board-certified child and adolescent psychiatrists with the participants and their parents to adhere closely to common clinical practice. See online Supplementary Material for information on recruitment, consent/assent, and exclusion criteria.

### Measures

#### The PA task

**Fig. 1.**
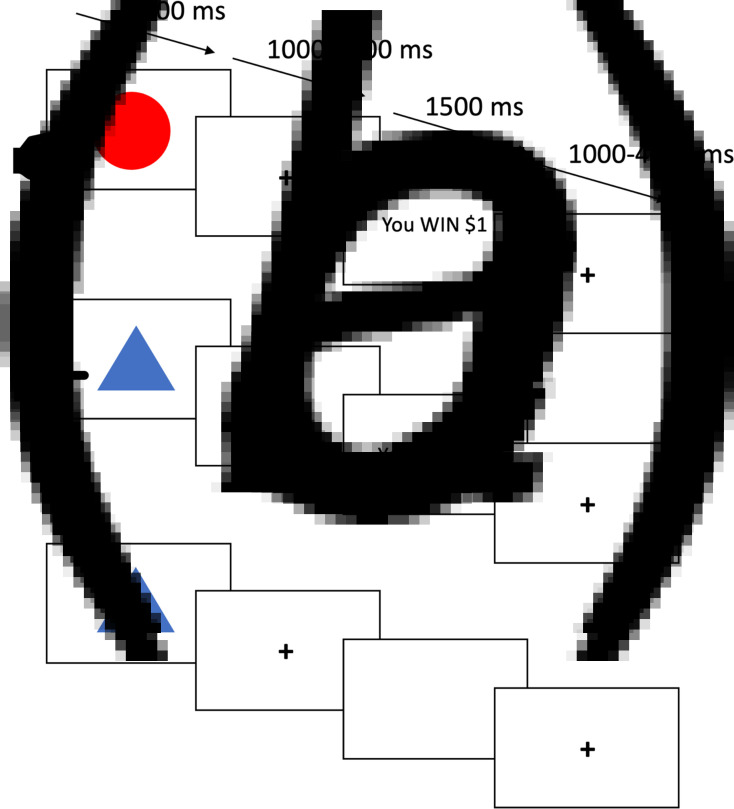
Three trials from the passive avoidance task. (A) The participant responds and receives a reward. (B) The participant responds and receives ‘punishment’. (C) The participant avoids responding and receives no feedback.

In the PA task (Kosson et al., 2006; Newman & Kosson, 1986), participants were presented with one of four shapes on each trial. They were told that some shapes were more likely to be associated with reward while others were more likely to be associated with loss. Their goal was to learn which stimuli were ‘good’ (i.e. more often than not engendering reward) and should be responded to and which were ‘bad’ (i.e. more often than not engendering loss) and should be avoided. Each trial began with the presentation of a shape (1500 ms). During this period, the participant had to decide whether to respond by button press or avoid responding. There was then a jittered fixation point interval (1000–4000 ms), followed, if the participant had responded to the stimulus, by either reward or punishment feedback (1500 ms). If the participant had not responded, the screen would be black for the 1500 ms feedback period. There was then a second jittered fixation point interval (1000–4000 ms) before the next trial began. Two of the four shapes paradigm typically yielded a virtual monetary reward (80% probability of winning $1 or $5 on each trial, see Fig. 1*A*) if responded to. The other two shapes typically yielded a virtual monetary punishment (80% probability of losing $1 or $5 on each trial, see Fig. 1*B*) if responded to. Shapes were presented in a randomized order. There were 27 trials for each shape, totaling 108 trials.

#### Inventory of Callous-Unemotional Traits (ICU)

The ICU is a 24-item self-report scale designed to assess CU traits in youth. The construct validity of the ICU has been supported in community and juvenile justice samples (Essau, Sasagawa, & Frick, 2006; Frick, 2004; Kimonis et al., 2008).

#### Affective Reactivity Index (ARI)

The ARI is a seven-item self-report questionnaire that assesses the youth's irritability during the preceding 6 months (six symptom items and one function impairment item). Prior work has indicated that the ARI is a reliable and valid measure of irritability in youth (Stringaris et al., 2012).

#### Strengths and Difficulties Questionnaire (SDQ)

The SDQ is a 25-item parent-report questionnaire which measures child emotional and behavioral problems (Goodman, 1997). The SDQ comprises five scales of five items each. The conduct problems (SDQ-CP) subscale was used to measure conduct problems (range 0–10). The SDQ shows moderate test-retest reliability (Yao et al., 2009) and good concurrent validity (Muris, Meesters, & van den Berg, 2003).

### MRI parameters

All data were collected on a 3 T Siemens Skyra scanner. A total of 313 functional images were taken with a T2* weighted gradient echo planar imaging (EPI) sequence (repetition time = 2500 ms; echo time = 27 ms; 240 mm field of view; 94×94 matrix; 90° flip angle). Whole-brain coverage was obtained with 43 axial slices (thickness = 2.5 mm; voxel size = 2.6 × 2.6 × 2.5 mm^3^). A high-resolution T1 anatomical scan (MP-RAGE, repetition time = 2200 ms; echo time = 2.48 ms; 230 mm field of view; 8° flip angle; 256 × 208 matrix; thickness = 1 mm; voxel size = 0.9 × 0.9 × 1 mm^3^) along with the EPI data set was obtained covering the whole brain with 176 axial slices.

### Functional MRI analysis: data preprocessing and individual-level analysis

Functional MRI data were preprocessed and analyzed using Analysis of Functional NeuroImages software (Version 19.2.10, AFNI; Cox, 1996). Skull-stripped anatomic images were normalized via non-linear registration to AFNI's TT_N27 Talairach-space template. EPI images were processed by removing premagnetization volumes, despiking, correcting slice-timing, aligning all EPI volumes to a reference volume and to the normalized anatomic image, spatially smoothing with a 6 mm FWHM Gaussian kernel, and voxelwise intensity scaling to a mean of 100.

Afterwards, four indicator regressors were generated: one for approached stimuli, one for avoided stimuli, one for reward feedback, and one for punishment feedback. Volumes were censored if there was >0.5 mm motion across adjacent volumes. There were no significant correlations between ARI scores and censored volumes, average motion per volume, or maximum displacement during scanning within the final sample (*r*'s = 0.093–0.097, *p* > 0.20). There were no significant correlations between ICU scores and censored volumes, average motion per volume or maximum displacement (*r* = −0.113 to −0.031, *p* > 0.10).

Conditions were modeled with a *γ* variate hemodynamic response function to account for the slow hemodynamic response. Generalized linear model (GLM) fitting was performed with the four regressors listed, six motion regressors, and a regressor modeling baseline drift (-polort 4) producing a *β***-**coefficient for each voxel and regressor.

### Statistical analyses

#### Clinical data

Potential group differences in age, IQ, and sex as well as expected group differences in ICU, ARI, and SDQ-CP scores were examined via independent samples *t* tests. Zero-order correlation analyses between these measures across both the participants with CD and the whole sample were calculated.

#### Behavioral data

To examine group differences in behavioral data, a one-way (Group: CD, TD) repeated measures analyses of variance (ANOVA) was conducted on the response type data (Response Type: Hit, Correct Avoidance). ‘Hits’ represent (correct) responses to stimuli that were probabilistically associated with reward. ‘Correct Avoidances’ represent (correctly) withheld responses to stimuli that were probabilistically associated with punishment. A one-way repeated measure analysis of covariance (ANCOVA) on the response type data using ICU and ARI scores as continuous covariates was conducted for the participants with CD.

#### fMRI data

To examine group differences in BOLD response data, a 2 (Group: CD, TD) by 2 (Feedback: Reward, Punishment) repeated measures ANOVA was conducted on the BOLD response data using 3dMVM.

To examine associations between ICU and ARI scores and dysfunction within brain regions responsive to Feedback, we ran a one-way (Feedback: Reward, Punishment) repeated measures ANCOVA for the CD participants on the BOLD response data with ICU and ARI scores as continuous covariates using 3dMVM. To reduce skewness and kurtosis (and thus disproportionate influence on coefficient estimates of data points in the tails/outliers), a Rankit transformation was applied to ICU and ARI scores (Bliss, Greenwood, & White, 1956). Pre-transformation, skewness and kurtosis were 1.450 and 1.456 for the ARI and 0.463 and 0.294 for the ICU. Following transformation, these were 0.425 and −0.508 and −0.003 and −0.102, respectively. The Rankit-transformed ICU and ARI scores were then *z*-scored, and these values were used as continuous covariates in all ANCOVA analyses.

We chose to use transformed ARI and ICU scores because of concerns that participants with outlier scores on these variables might disproportionately influence the results. Note all ANCOVAs conducted on the BOLD response data within AFNI assessed inter-covariate interactions.

Follow-up partial correlations were performed within RStudio and Steiger's *Z*-tests within a freely available online tool (http://quantpsy.org/corrtest/corrtest2.htm). Steiger's *Z*-tests (Steiger, 1980) were used to estimate the difference of correlation strengths of ICU and ARI with clinical variables. In order to facilitate future meta-analytic work, effect sizes for all clusters are reported.

Correction for multiple comparisons was performed using a spatial clustering operation in AFNI's 3dClustSim utilizing the autocorrelation function (-acf) with 10 000 Monte Carlo simulations for the whole-brain analysis. Spatial autocorrelation was estimated from residuals from the individual-level GLMs. The initial threshold was set at *p* = 0.001. This process yielded an extant threshold of *k* = 23 voxels for the whole brain (multiple comparison corrected *p* < 0.05).

### Follow-up analyses

#### Psychiatric comorbidity

Given that a significant number of the participants with CD were co-morbid for major depressive disorder (MDD) and generalized anxiety disorder (GAD), our ANOVA analysis was repeated twice: once excluding participants with an MDD diagnosis (*N* = 18) and once excluding participants with a GAD diagnosis (*N* = 31). Rates of ADHD were extremely high in our participants with CD (80%; see Table 1). Similarly, ADHD symptom severity as indexed by the Conners ADHD scale was highly correlated with CD diagnostic status (*r* = 0.794). Given consequent collinearity concerns, we could not disentangle this potential confound with respect to the Group results. However, the association of ADHD symptom severity within the participants with CD with ARI/ICU scores was substantially lower (*r* = 0.173 and 0.104, respectively). As such, our ANCOVA analysis was repeated with an additional covariate (ADHD severity).

#### Prescribed medications

Given that a significant number of the participants with CD were prescribed medications (e.g. anti-psychotic medications, SSRIs, and stimulants), our ANOVA analysis was repeated three times: once excluding participants prescribed antipsychotic medications (*N* = 14), once excluding participants prescribed SSRIs (*N* = 24), and once excluding participants prescribed stimulants (*N* = 17).

#### Single covariate analyses

To ensure that inter-covariate suppressor effects could not be obscuring any potential individual covariate associations, our symptom severity ANCOVA analysis was repeated twice (once with Rankit-transformed, *z*-scored ICU scores as the covariate and once with Rankit-transformed, *z*-scored ARI scores as the covariate).

#### Whole sample analysis

While our main goal with the covariate analyses was to determine the extent to which CU or irritability severity within the sample with CD was associated with atypical responsiveness, we also repeated the one-way (Feedback: Reward, Punishment) repeated measures ANCOVA on the BOLD response data with ICU and ARI scores as continuous covariates for the whole sample.

#### Raw ICU and ARI analysis

The ANCOVA analysis using the Rankit-transformed *z*-scored ICU and ARI scores as continuous covariates may raise the concern about generalization in other studies where the ICU and ARI scores have not been transformed. Therefore, the ANCOVA with the participants with CD was repeated using raw ICU and raw ARI scores as continuous covariates.

## Results

### Clinical data

#### Group differences

As expected, participants with CD scored significantly higher than TD participants on the SDQ-CP, ICU, and ARI. There were no group differences in ages, sex, or IQ; see Table 1.

#### CD sample results

Within the CD group, ICU and ARI were significantly positively correlated (*r* = 0.299, *p* < 0.01). ARI was negatively associated with IQ (*r* = −0.295, *p* < 0.01) but not with age or sex. ICU was not associated with age, IQ, or sex. Neither traits were significantly correlated with the prescription of antipsychotic, stimulant, or SSRI medications.

Whole sample correlation results are displayed in online Supplementary Table S2.

### Behavioral data

The group-based ANOVA revealed a significant main effect of response type [*F*_(1,176)_ = 52.259; *p* < 0.001; *η*^2^ = 0.229]. Participants made more ‘Hit’ correct responses than ‘Correct Avoidances’ (*M*_Hit_ = 78.2%, *M*_Correct Avoidance_ = 64.9%). There was also a significant Group main effect [*F*_(1,176)_ = 6.177, *p* *=* 0.014; *η*^2^ = 0.034]: participants with CD made significantly less correct responses than TD participants (*M*_CD_ = 68.6%, *M*_TD_ = 78.2%).

The ANCOVA conducted on the participants with CD revealed the same significant main effect of response type [*F*_(1,90)_ = 53.024; *p* < 0.001; *η*^2^ = 0.243]. However, there were no significant effects of ICU or ARI score or significant interactions of these variables with response type (*F* = 0.005–1.108; *p* = 0.294–0.945; *η*^2^ = 0.000–0.007).

### fMRI data

#### Group-based ANOVA

Regions displayed a significant main effect of Feedback (online Supplementary Table S3) and Group-by-Feedback interaction (Table 2).

**Table 2.** Regions showing significant Group-by-Feedback interactions from the Group-based (CD, TD) ANOVA analysis and regions showing significant ICU-by-Feedback interactions from the ANCOVA analysis conducted with the participants with CD

Region	BA	Voxels	*X*	*Y*	*Z*	*F*-value	*η*^2^
*Group-by-Feedback*							
L caudate		52	−4	8	−1	34.527	0.164
R caudate		30	11	2	23	22.081	0.111
L vmPFC***	32/10	37	−10	44	−1	14.548	0.076
*ICU-by-Feedback*							
R lateral frontal cortex extending to dmFC	8/9/32	74	20	35	41	25.689	0.218
L lateral frontal cortex	8	57	−20	23	50	22.578	0.197
R superior frontal gyrus	10	50	23	56	17	20.592	0.183
L superior temporal gyrus/superior parietal lobule	39/7	98	−34	−55	29	27.509	0.230
R precentral gyrus	6	30	41	−10	41	26.875	0.226
R caudate		28	17	17	23	26.083	0.221
R vmPFC***	32/10	136	11	29	−4	27.333	0.231

Coordinates based on the Tournoux and Talairach standard brain template; BA, Brodmann's Area; ***vmPFC results recorded at the initial threshold of *p* < 0.005.

**Fig. 2.**
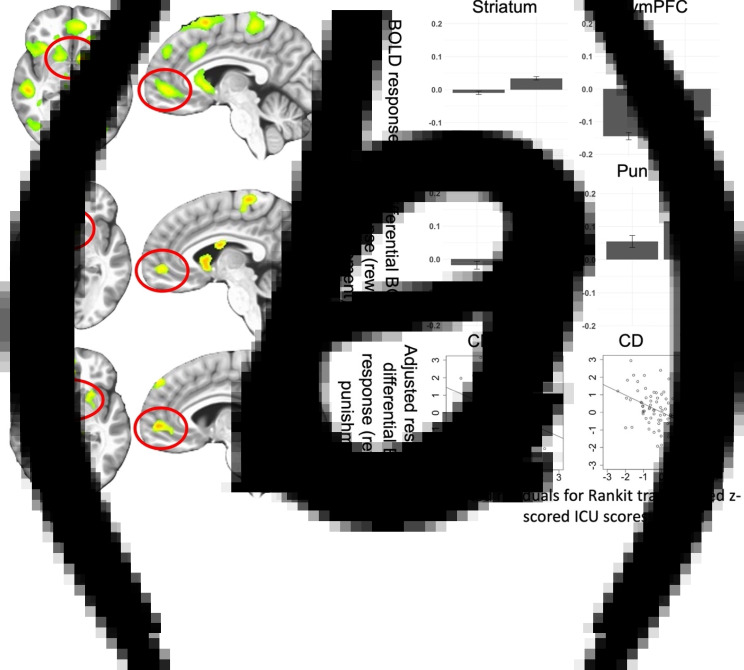
(A) Regions showing the main effect of Feedback (Reward, Punishment) from the Group-based ANOVA. Bar graphs show BOLD responses within the striatum and vmPFC for all participants in the study. (B) Regions showing Group-by-Feedback interaction from the Group-based ANOVA.*Bar graphs show differential BOLD responses to reward *v*. punishment within the striatum and vmPFC for participants with CD and TD participants. (C) Regions showing ICU-by-Feedback interaction from the main ANCOVA.*Scatterplots depict the partial correlations and adjusted residuals for the striatum and vmPFC. Adjusted residuals for the Rankit-transformed *z*-scored ICU scores (*x*-axis) are plotted against adjusted residuals for the differential BOLD responses to reward *v*. punishment. Key to Fig. 2: Pun, punishment; Rew, reward; CD, conduct disorder; TD, typically developing; *figures shown at *p* < 0.005 to illustrate vmPFC.

*Main Effect of Feedback* was observed in regions including the bilateral striatum and left vmPFC [*F*_(1,176)_ = 73.941 and 32.976; *p* < 0.001; *η*^2^ = 0.296 and 0.158, respectively]. Participants had significantly higher BOLD responses to Reward relative to Punishment; see online Supplementary Table S3 and Fig. 2*A*.

A *Group-by-Feedback Interaction* was observed within the bilateral striatum [BA 25 and 24, *F*_(1,176)_ = 34.527 (left) and 22.081(right); *p* < 0.001; *η*^2^ = 0.164 and 0.111]. Participants with CD had significantly less differential BOLD responses to Reward relative to Punishment [*t*(176) = −5.876 and −4.699, *p* < 0.001] compared to TD; see Fig. 2*B* and Table 2.

#### CU and irritability severity ANCOVA for participants with CD

**Fig. 3.**
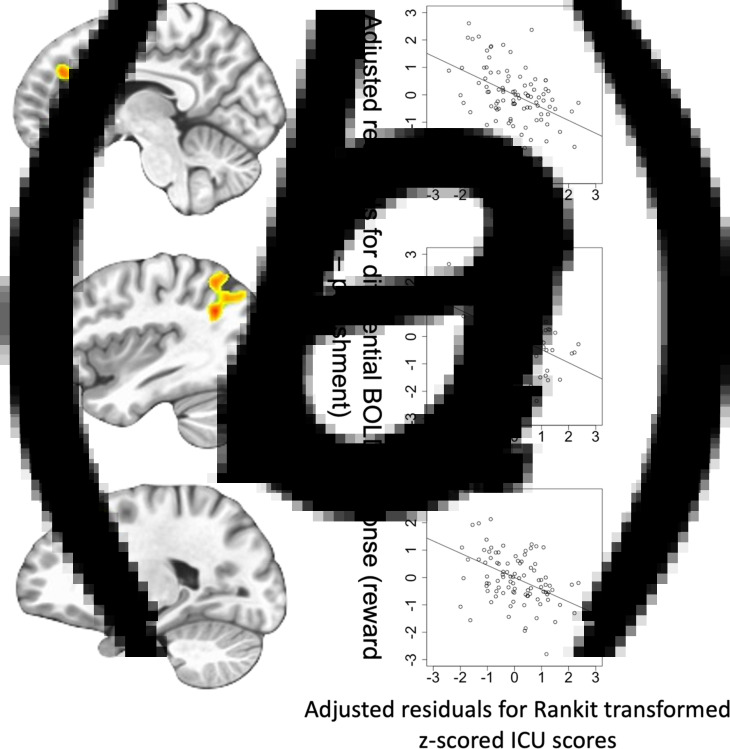
Regions showing ICU-by-Feedback interaction: (A) rostromedial frontal cortex; (B) left superior temporal gyrus/superior parietal lobule; (C) lateral frontal cortex. Scatterplots depict the adjusted residuals for the Rankit-transformed *z*-scored ICU scores (*x*-axis) against adjusted residuals for the differential BOLD responses to reward *v*. punishment.

The ANCOVA revealed regions displaying significant CU traits-by-Feedback interactions (see Fig. 2*C*, Fig. 3, and Table 2). Significant main effects are reported in online Supplementary Table S4. No regions showed significant ARI-by-Feedback interactions even at more lenient statistical thresholds (*p* < 0.005).

*CU traits-by-Feedback Interactions* were observed in regions including the right middle frontal/rostromedial cortex, left lateral frontal gyrus, right superior frontal gyrus, right precentral gyrus, left superior temporal gyrus/superior parietal lobule, and right caudate [BA 39, 8, 10, 6, and 7, *F*_(1,90)_ = 20.592–27.509; *p* < 0.001; *η*^2^ = 0.183–0.230]. In these regions, CU traits were negatively associated with differential BOLD responses to Reward relative to Punishment (Steiger's *Z* = −4.807 to −3.848, *p* < 0.001).

### Follow-up analyses

#### Potential confounds: psychiatric co-morbidities

Given the significant associations of CD with other diagnoses (see Table 1), our ANOVA analysis was repeated twice, once excluding participants with an MDD diagnosis (*N* = 18) and once excluding participants with a GAD diagnosis (*N* = 31). Both ANOVAs largely replicated the results of our main analysis (for full details, see online Supplementary Table S6). Similarly, our additional ANCOVA analysis with the participants with CD additionally including ADHD severity largely replicated the results of our main analysis (for full details, see online Supplementary Table S7).

#### Prescribed medications

The follow-up ANOVAs each separately excluding participants prescribed antipsychotic medications (*N* = 14), participants prescribed SSRIs (*N* = 24), and participants prescribed stimulants (*N* = 17) also largely replicated the results of our main analysis (for full details, see online Supplementary Table S6).

#### Single covariate analyses

Analyses examining the ICU and ARI separately (to ensure that inter-covariate suppressor effects could not be obscuring any potential individual covariate associations) largely replicated our BOLD response main results. The ICU ANCOVA revealed significant comparable ICU-by-Feedback interactions in proximal regions (see online Supplementary Table S8). The ARI ANCOVA failed to identify regions showing significant ARI-by-Feedback interactions.

#### Whole sample analysis

The covariate analysis for the whole sample largely replicated the results of the sample with CD analysis albeit with activations at smaller extent thresholds. Regions showing significant ICU-by-Feedback interactions are reported in online Supplementary Table S9. No regions showed significant ARI-by-Feedback interactions.

#### Raw ICU and ARI analysis

The ANCOVA analysis with raw ICU and ARI scores as covariates revealed very similar results to that with Rankit-transformed *z*-scored ICU and ARI scores. The detailed results are reported in the online Supplementary Table S5.

## Discussion

The goals of this study were to determine: (i) responsiveness to reward relative to punishment in participants with CD compared to TD adolescents; and (ii) the extent of association between symptoms related to CU traits or irritability and dysfunctional differential reward-punishment responsiveness in adolescents with CD. The two main results of this study were: (i) that adolescents with CD showed reduced differential reward-punishment responsiveness relative to TD adolescents; and (ii) CU traits, but not irritability, were associated with reduced differential reward-punishment responsiveness in a number of frontal regions as well as the striatum.

There are a number of core regions implicated in responding to reward and punishment including the striatum, vmPFC, PCC, AIC, and ACC (Clithero & Rangel, 2014; O'Doherty et al., 2017; Schoenbaum et al., 2011). All of these regions, except PCC, showed differential reward-punishment responsiveness in the sample as a whole (see the main effect of feedback; online Supplementary Table S3 and Fig. 2*A*). Previous work has reported reduced reward responses in patients with CD relative to TD adolescents in regions within the striatum (Cohn et al., 2015; Hwang et al., 2018; White et al., 2013) and vmPFC (Finger et al., 2011; Rubia et al., 2009). In line with predictions, the participants with CD in the current sample showed significantly reduced reward-punishment differential responsiveness relative to TD adolescents.

The second goal of the current paper was to determine the extent of association between symptoms related to CU traits or irritability and dysfunctional differential reward-punishment responsiveness in adolescents with CD. Our data indicated that the severity of CU traits within the participants with CD was negatively associated with differential reward-punishment responsiveness within a number of regions including regions responsive to reinforcement information such as the striatum (cf. Clithero & Rangel, 2014; O'Doherty et al., 2017; Schoenbaum et al., 2011), as seen in the group-based ANOVA. In addition, these included regions implicated in attention (e.g. dorsomedial, lateral frontal, and parietal cortices; Desimone & Duncan, 1995; Kastner & Ungerleider, 2000; Miller & Buschman, 2013), potentially reflecting a reduced attentional response to reinforcement information as a function of CU traits. Previous work in clinical populations has been relatively inconsistent with respect to the relationship between CU traits and reward responsiveness (Byrd et al., 2018; Cohn et al., 2015; Schwenck et al., 2017; White et al., 2013). However, several of these studies have involved relatively small *N*s (Byrd et al., 2018; Schwenck et al., 2017; White et al., 2013) or non-clinical samples (Murray, Shaw, Forbes, & Hyde, 2017). Indeed, the two larger *N* studies (Cohn et al., 2015; Veroude et al., 2016) both reported negative associations between CU traits and reward responsiveness (though in the Cohn et al. study, this was within the amygdala not striatum). In short, we believe the current data, in conjunction with previous work, indicate that CU traits may be negatively associated with reward responsiveness.

There were no indications of an association between irritability and reward responsiveness in the current study irrespective of whether the association was examined within the participants with CD or the entire sample. Moreover, the lack of association was also seen at more lenient statistical thresholds (*p* < 0.005). There has been one previous report of increased striatal responsiveness to reward as a function of the level of irritability in adolescents from intervention-seeking families in the community (Kryza-Lacombe et al., 2021). However, work examining frustration in youth with SMD has previously reported typical responses to reward (Deveney et al., 2013) – a finding consistent with the present results. Given the inconsistency, future work will be necessary to address the issue. In addition, there have been reports of both increased and decreased responses within dmFC, dlPFC, AIC/iFC, and striatum to negative feedback or information on the incorrectness of responses (Adleman et al., 2011; Deveney et al., 2013; Rich et al., 2011; Tseng et al., 2019). The latter findings allow the speculation that there may be associations between irritability and atypical responses to reinforcement information but these may be more marked for punishment (particularly punishment indicating the incorrectness of responses). Future work will need to specifically examine this issue.

Several caveats should be considered with respect to the current results. First, the participants with CD showed significant psychiatric comorbidities; the vast majority presented with ADHD and significant numbers presented with both MDD and GAD. All three of these diagnoses have also been associated with reduced reward responsiveness (O'Callaghan & Stringaris, 2019; Plichta & Scheres, 2014; White et al., 2017). However, the main findings remained present following the removal of the participants with MDD and GAD. As such, it appears unlikely that the current results can be attributed to pathology underlying the diagnosis of MDD and GAD. However, the same analysis cannot be conducted with respect to ADHD. In the current sample, 80% of the participants with CD were co-morbid for ADHD. As such, the current results could be attributed to the pathology underlying the diagnosis of ADHD. However, it is important to note here that the severity of CU traits was related to the dysfunction shown by the participants with CD. In contrast, CU traits have not been associated with the most common forms of pathology associated with ADHD (e.g. Hwang et al., 2016). In short, we believe that reduced striatal responding to reward may be a more general pathological feature that is associated with the exacerbation of a number of psychiatric phenotypes (though not irritability) that show decision-making impairments. Second, we did not implement structured or semi-structured diagnostic interview. However, it is important to note that our adolescents with CD showed markedly more severe conduct problems on the SDQ as well as the ARI and ICU (see Table 1). Moreover, it is important to note that the main goal of this work was to investigate the associations of specific symptom types (irritability and CU traits) across a population showing very significant conduct problem. Third, the participants with CD were prescribed a number of psychiatric medications. However, the analyses removing the participant prescribed anti-psychotic medications, SSRIs, or stimulants did not significantly change our core results. Fourth, given significant skewness and kurtosis in the pre-transformation ARI scores, these were Rankit-transformed to reduce the possibility of a disproportionate influence on the coefficient estimates of data points in the tails/outliers. However, such an approach may lead to difficulties in generalization with respect to other studies where the ARI scores have not been transformed. However, it is important to note that the results of the ANCOVA analysis featuring raw ARI and ICU scores largely mirrored the main ARI/ICU ANCOVA analysis (see online Supplementary Table S5). Fifth, the current study revealed dysfunction in the differential response to received rewards and punishments. It did not investigate responses to rewards and punishment as a function of expectations based on previous reinforcement history (i.e. prediction error signaling). However, pilot analyses of BOLD response data on the current PA task in healthy participants revealed that this task implementation was not optimized to reveal a strong prediction error signal. Future computational modeling-based work with other tasks will investigate this issue.

In conclusion, adolescents with CD showed reduced differential reward-punishment responsiveness within the striatum and that the severity of this dysfunction within the cases with CD was associated with the level of CU traits. The data do not support suggestions relating CD and/or CU traits to either increased responsiveness to reward (e.g. Buckholtz et al., 2010; Frick et al., 2014) or an attentional response bias to reward-related information (Newman, 1998) – indeed, there was reduced differential recruitment of regions implicated in attention in response to reward *v.* punishment as a function of CU traits. Instead, the current data are consistent with a compromised response to reward *v.* punishment information that may disrupt learning, leading to poorer decision-making that may particularly manifest as CU traits.

## Supplementary material

For supplementary material accompanying this paper visit https://doi.org/10.1017/S0033291721003500. click here to view supplementary material
