# Supplementary material for: Dysfunction in differential reward-punishment responsiveness in conduct disorder relates to severity of callous-unemotional traits but not irritability
Source: Psychol Med. 2021 Sep 1;53(5):1870–80. doi: 10.1017/S0033291721003500 (PMC8885913; doi:10.1017/S0033291721003500)
Supplement: Supplementary file 1 [file S0033291721003500sup001.docx]

**Supplemental Material**

**Table of Contents**

1. **Consenting and Assenting Procedures**
2. **Exclusion criteria**
3. **Analysis examining differential reinforcement responding in individuals with high vs. low ICU scores**
4. **Results of the 2 (Group: CD, TD) by 2 (Feedback: Reward, Punishment) repeated measures ANCOVA on the BOLD response data with correct response rate as a covariate.**
5. **Table S1: Regions showing Group-by-Feedback interactions from the Group-based (high ICU scoring, low ICU scoring) ANOVA analysis conducted with the participants with CD.**
6. **Table S2. Correlations of demographic and clinical variables for the whole sample.**
7. **Table S3. Regions showing a significant main effect of Feedback in the Group-based (CD, TD) ANOVA analysis.**
8. **Table S4. Regions showing significant main effects of ICU and Feedback from the ANCOVA conducted on the participants with CD.**
9. **Table S5. Regions showing ICU-by-Feedback interactions from the ANCOVA analysis with raw ICU and raw ARI scores as continuous covariates for the CD participants.**
10. **Table S6. Caudate and vmPFC (at *p* < 0.005) showing Group-by-Feedback interaction in the follow-up ANOVAs.**
11. **Table S7. Regions showing ICU-by-Feedback interactions from the ANCOVA analysis including ADHD severity as well as ICU and ARI scores as continuous covariates for the CD participants.**
12. **Table S8. Regions showing ICU-by-Feedback interactions from the ANCOVA analysis with only ICU score included as a covariate for the CD participants.**
13. **Table S9. Regions showing ICU-by-Feedback interactions from the ANCOVA analysis conducted on the whole sample.**
14. **Consenting and Assenting Procedures**

The Boys Town National Research Hospital institutional review board approved this study. A doctoral level researcher or a member of the clinical research team obtained written informed consent and assent. In all cases, youth had the right to decline participation at any time before or during the study. With respect to community participants, informed consent was obtained from the youths’ parents/legal guardians at the beginning of the on-site screening. At this time, the consent document was reviewed in detail and the parents/legal guardians had the opportunity to have their questions answered before being asked to sign the consent form. After that, informed assent was obtained from the youths themselves. This procedure differed slightly for youth recruited from the Boys Town campus. Consent was typically obtained from parents during or shortly after the child’s arrival at Boys Town. Assent was obtained from the youth in a separate session, 5-10 days after parental consent had been obtained.

1. **Exclusion Criteria**

Exclusion criteria included IQ < 75 assessed with the Wechsler Abbreviated Scale of Intelligence (WASI two-subtest form; Wechsler, 2011), pregnancy, non-psychiatric medical conditions that require the use of medication that may have psychotropic effects (e.g., beta blockers or steroids), current psychosis, pervasive developmental disorders, Tourette’s disorder, neurological disorders, presence of metallic objects in the body (e.g., metal plates, pacemakers, etc.), and claustrophobia. Current psychiatric conditions (other than psychotic disorders or pervasive developmental disorders) were not exclusionary. Use of psychotropic medications for psychiatric indications (e.g., stimulants, selective serotonin reuptake inhibitors) were not exclusory. However, participants on stimulant medication were asked to withhold medication on the morning of the scan.

1. **Analysis examining differential reinforcement responding in individuals with CD who showed high vs. low ICU scores**

While clinical cut-offs for the self-report Inventory of Callous-Unemotional traits have not been formally specified, there have been suggestions that a cut-off of 29 can be used (Kemp et al., 2020). We thus conducted an analysis with the 24 participants with CD with an ICU score > 29 and a comparable number (*N* = 19) of participants with CD whose ICU score < 19. Conducting a 2 (Group: High, Low ICU scoring individuals) by 2 (Feedback: Reward, Punishment) ANOVA revealed that very proximal regions to those previously identified as showing ICU-by-Feedback interactions in our main ANCOVA showed Group-by-Feedback interactions in this analysis (see Table S1). Given the considerably smaller *N* in this analysis, results are reported at *p* < 0.005.

1. **Results of the 2 (Group: CD, TD) by 2 (Feedback: Reward, Punishment) repeated measures ANCOVA on the BOLD response data with correct response rate as a covariate.**

Similar to the main ANCOVA analysis, this revealed Group-by-Feedback interactions in bilateral caudate (xyz = -10, 14, 17 & 11, 2, 23, 38 and 28 voxels respectively) and in left vmPFC at *p* < 0.005 (xyz = -10, 44, -1; 26 voxels).

Table S1. Regions showing Group-by-Feedback interactions from the Group-based (high ICU scoring, low ICU scoring) ANOVA analysis conducted with the participants with CD.

| **REGION** | **BA** | **Voxels** | **X** | **Y** | **Z** |
| --- | --- | --- | --- | --- | --- |
|  |  |  |  |  |  |
| ***Group-by-Feedback*** |  |  |  |  |  |
|  |  |  |  |  |  |
| R lateral frontal cortex | 8/9/32 | 36 | 20 | 35 | 38 |
| L lateral frontal cortex | 8 | 17 | -19 | 23 | 53 |
| R superior frontal gyrus | 10 | 17 | 23 | 56 | 17 |
| L superior temporal gyrus/superior parietal lobule | 39/7 | 187 | -34 | -52 | 47 |
| R precentral gyrus | 6 | 55 | 41 | -10 | 41 |
| R caudate |  | 28 | 20 | 14 | 17 |
| R vmPFC | 32/10 | 11 | 8 | 26 | -7 |
|  |  |  |  |  |  |
|  |  |  |  |  |  |

Note: Coordinates based on the Tournoux & Talairach standard brain template, BA = Brodmann’s Area.

Table S2. Correlations of demographic and clinical variables for the whole sample.

|  | **Correlation with ARI for the whole group** | **Correlation with ICU for the whole group** | **Steiger's Z** | ***p* value** |
| --- | --- | --- | --- | --- |
| **Age** | 0.124 | 0.165* | -0.541 | 0.589 |
| **IQ** | -0.240** | -0.121 | -1.585 | 0.113 |
| **ARI** | -- | 0.483** | -- | -- |
| **ICU** | 0.483** | -- | -- | -- |
| **SDQ-CP** | 0.513** | 0.542** | -0.468 | 0.639 |
| **RPRS Total** | 0.406** | 0.436** | -0.443 | 0.658 |
| **RPRS Reactive** | 0.406** | 0.411** | -0.519 | 0.604 |
| **RPRS Proactive** | 0.365** | 0.426** | -0.886 | 0.375 |
| **Conners (ADHD)** | 0.384** | 0.323** | 0.865 | 0.387 |
|  |  |  |  |  |
| **Male** | -0.038 | 0.152 | -1.949 | 0.051 |
|  |  |  |  |  |
| **MDD** | 0.323** | 0.258** | 0.262 | 0.793 |
| **GAD** | 0.352** | 0.278** | 0.412 | 0.680 |
| **ADHD** | 0.473** | 0.416** | 0.568 | 0.569 |
| **CD** | 0.507** | 0.496** | 0.13 | 0.897 |
|  |  |  |  |  |
| **Antipsychotic** | 0.127 | 0.128 | -0.004 | 0.997 |
| **Stimulant** | 0.110 | 0.088 | 0.097 | 0.922 |
| **SSRI** | 0.074 | 0.189* | -0.486 | 0.627 |

Note: MDD: Major Depressive Disorder; GAD: Generalized Anxiety Disorder; ADHD: Attention Deficit Hyperactivity Disorder; CD: Conduct Disorder; *p* = two-tailed significance level of the Steiger’s Z calculation (i.e., Whether there were significant differences in correlation strength between the ARI scores and ICU scores). * *p* < 0.05. ** *p* < 0.01.

Table S3. Regions showing a significant main effect of Feedback in the Group-based (CD, TD) ANOVA analysis.

| **REGION** | **BA** | **Voxels** | **X** | **Y** | **Z** | ***F*-value** | ***η*²** |
| --- | --- | --- | --- | --- | --- | --- | --- |
|  |  |  |  |  |  |  |  |
| ***Feedback*** |  |  |  |  |  |  |  |
|  |  |  |  |  |  |  |  |
| L/R caudate/nucleus accumbens | -- | 664 | 10 | 8 | -1 | 73.941 | 0.296 |
| L vmPFC | 32/10 | 156 | -10 | 41 | -1 | 32.976 | 0.158 |
| R dmFC | 6/8/32 | 485 | 5 | 8 | 59 | 42.197 | 0.193 |
| L medial frontal gyrus | 8 | 88 | -16 | 11 | 44 | 27.212 | 0.134 |
| L superior frontal gyrus | 10 | 29 | -13 | 56 | 20 | 19.479 | 0.100 |
| R anterior insula cortex | 13 | 339 | 35 | 11 | 5 | 42.968 | 0.196 |
| L anterior insula cortex | 13 | 337 | -28 | 20 | -4 | 46.283 | 0.208 |
| R inferior parietal lobule | 40 | 64 | 56 | -40 | 26 | 23.418 | 0.117 |
| L supramarginal gyrus/inferior parietal lobule | 40 | 45 | -55 | -46 | 26 | 24.541 | 0.127 |
| R postcentral gyrus | 40 | 27 | 41 | -34 | 50 | 17.575 | 0.091 |
| R precentral gyrus | 3 | 536 | 11 | -31 | 68 | 34.992 | 0.166 |
| R superior temporal gyrus | 38 | 125 | 50 | 11 | -25 | 42.696 | 0.195 |
| L superior temporal gyrus | 38 | 54 | -49 | 11 | -13 | 29.138 | 0.142 |
| L superior temporal gyrus | 41/42 | 36 | -58 | -16 | 8 | 25.875 | 0.128 |
| R middle temporal gyrus | 21 | 104 | 50 | -28 | -1 | 37.741 | 0.177 |
| R cuneus | 17 | 89 | 17 | -91 | 8 | 25.358 | 0.126 |
| R middle occipital gyrus | 19 | 57 | 41 | -67 | -7 | 24.398 | 0.122 |
| R thalamus | -- | 32 | 5 | -25 | 2 | 38.643 | 0.180 |
|  |  |  |  |  |  |  |  |
|  |  |  |  |  |  |  |  |

Note: Coordinates based on the Tournoux & Talairach standard brain template, BA= Brodmann’s Area.

Table S4. Regions showing significant main effects of ICU and Feedback from the ANCOVA conducted on the participants with CD.

| **REGION** | **BA** | | **Voxels** | | **X** | | **Y** | | **Z** | | ***F-* value** | | ***η*²** | | |
| --- | --- | --- | --- | --- | --- | --- | --- | --- | --- | --- | --- | --- | --- | --- | --- |
|  |  | |  | |  | |  | |  | |  | |  | | |
| ***ICU*** | |  | |  | |  | |  | |  | |  | |  |  |
|  | |  | |  | |  | |  | |  | |  | |  |  |
| L/R paracentral lobule | | 31 | | 32 | | 2 | | -19 | | 44 | | 19.318 | | 0.174 |  |
| R precuneus | | 7 | | 27 | | 17 | | -76 | | 47 | | 20.161 | | 0.180 |  |
|  |  | |  | |  | |  | |  | |  | |  | | |
| ***Feedback*** |  | |  | |  | |  | |  | |  | |  | | |
|  |  | |  | |  | |  | |  | |  | |  | | |
| R superior frontal gyrus | 6 | | 177 | | 6 | | 8 | | 62 | | 41.424 | | 0.310 | | |
| R anterior insula cortex | 13 | | 57 | | 38 | | 11 | | 8 | | 26.799 | | 0.266 | | |
| L anterior insula cortex | 13 | | 274 | | -37 | | 14 | | 5 | | 48.735 | | 0.346 | | |
| L supramarginal gyrus/inferior parietal lobule | 40 | | 42 | | -58 | | -43 | | 26 | | 27.878 | | 0.233 | | |
| R middle temporal gyrus | 21 | | 50 | | 53 | | -25 | | -4 | | 36.774 | | 0.286 | | |
| R middle temporal gyrus | 21 | | 36 | | 50 | | 8 | | -25 | | 34.821 | | 0.275 | | |
| R lingual gyrus | 18 | | 23 | | 26 | | -55 | | 2 | | 22.324 | | 0.195 | | |
|  |  | |  | |  | |  | |  | |  | |  | | |

|  |  |  |  |  |  |  |  |
| --- | --- | --- | --- | --- | --- | --- | --- |

Note: Coordinates based on the Tournoux & Talairach standard brain template, BA= Brodmann’s Area.

Table S5. Regions showing ICU-by-Feedback interactions from the ANCOVA analysis with raw ICU and raw ARI scores as continuous covariates for the CD participants.

| **REGION** | **BA** | **Voxels** | **X** | **Y** | **Z** |
| --- | --- | --- | --- | --- | --- |
|  |  |  |  |  |  |
| ***ICU-by-Feedback*** |  |  |  |  |  |
|  |  |  |  |  |  |
| R lateral frontal cortex extending to dmFC | 8/9/32 | 48 | 20 | 32 | 41 |
| L lateral frontal cortex | 8 | 40 | -19 | 23 | 50 |
| R superior frontal gyrus | 10 | 30 | 23 | 56 | 17 |
| L superior temporal gyrus/superior parietal lobule | 39/7 | 28 | -34 | -61 | 38 |
| R precentral gyrus | 6 | 24 | 41 | -10 | 41 |
| R caudate |  | 25 | 20 | 17 | 14 |
| R vmPFC | 32/24 | 17 | 11 | 29 | -4 |
|  |  |  |  |  |  |
|  |  |  |  |  |  |

Note: Coordinates based on the Tournoux & Talairach standard brain template, BA = Brodmann’s Area.

Table S6. Caudate and vmPFC (at *p* < 0.005) showing Group-by-Feedback interaction in the follow-up ANOVAs.

| **REGION** | **BA** | **Voxels** | **X** | **Y** | **Z** | ***F*-value** | ***η*²** |
| --- | --- | --- | --- | --- | --- | --- | --- |
|  |  |  |  |  |  |  |  |
| ***Following exclusion of participants with MDD*** | | | | | | | |
| L caudate | -- | 19 | -7 | 8 | -1 | 21.416 | 0.119 |
| R caudate | -- | 23 | 11 | 2 | 23 | 23.188 | 0.128 |
| L vmPFC*** | 32/10 | 24 | -7 | 44 | -1 | 9.755 | 0.058 |
| ***Following exclusion of participants with GAD*** | | | | | | | |
| L caudate | -- | 27 | -1 | 8 | 5 | 26.307 | 0.154 |
| R caudate | -- | 23 | 11 | 2 | 23 | 23.188 | 0.128 |
| L vmPFC*** | 32/10 | 12 | -7 | 44 | -1 | 11.928 | 0.023 |
| ***Following exclusion of participants prescribed antipsychotic medications*** | | | | | | | |
| L caudate | -- | 22 | -10 | 14 | 17 | 23.210 | 0.128 |
| R caudate | -- | 31 | 11 | 2 | 23 | 21.382 | 0.119 |
| L vmPFC*** | 10 | 27 | -10 | 44 | -1 | 12.862 | 0.075 |
| ***Following exclusion of participants prescribed SSRIs*** | | | | | | | |
| L caudate | -- | 12 | -10 | -19 | 29 | 24.989 | 0.139 |
| R caudate | -- | 22 | 11 | 8 | 2 | 23.030 | 0.129 |
| L vmPFC*** | 32/10 | 34 | -7 | 44 | -1 | 13.743 | 0.081 |
| ***Following exclusion of participants prescribed stimulants*** | | | | | | | |
| L caudate | -- | 47 | -10 | 14 | 17 | 29.432 | 0.161 |
| R caudate | -- | 56 | 11 | 2 | 23 | 24.881 | 0.140 |
| L vmPFC*** | 32/10 | 60 | -4 | 50 | -1 | 16.520 | 0.097 |
|  |  |  |  |  |  |  |  |

Note: Coordinates based on the Tournoux & Talairach standard brain template, BA= Brodmann’s Area, *** vmPFC results recorded at initial threshold of *p* < 0.005.

Table S7. Regions showing ICU-by-Feedback interactions from the ANCOVA analysis including ADHD severity as well as ICU and ARI scores as continuous covariates for the CD participants.

| **REGION** | **BA** | **Voxels** | **X** | **Y** | **Z** |
| --- | --- | --- | --- | --- | --- |
|  |  |  |  |  |  |
| ***ICU-by-Feedback*** |  |  |  |  |  |
|  |  |  |  |  |  |
| R lateral frontal cortex | 8 | 8 | 20 | 38 | 47 |
| L lateral frontal cortex | 8 | 57 | -22 | 23 | 50 |
| R superior frontal gyrus | 10 | 10 | 20 | 53 | 20 |
| L superior temporal gyrus/superior parietal lobule | 39/7 | 35 | -34 | -61 | 38 |
| R caudate |  | 8 | 19 | 15 | 14 |
| R vmPFC*** | 32/10 | 109 | 8 | 35 | -10 |
|  |  |  |  |  |  |

Note: Coordinates based on the Tournoux & Talairach standard brain template, BA = Brodmann’s Area, ***vmPFC results recorded at initial threshold of *p* < 0.005.

Table S8. Regions showing ICU-by-Feedback interactions from the ANCOVA analysis with only ICU score included as a covariate for the CD participants.

| **REGION** | **BA** | **Voxels** | **X** | **Y** | **Z** | ***F*-value** | ***η*²** |
| --- | --- | --- | --- | --- | --- | --- | --- |
|  |  |  |  |  |  |  |  |
| ***ICU-by-Feedback*** |  |  |  |  |  |  |  |
|  |  |  |  |  |  |  |  |
| R lateral frontal cortex extending to rmFC | 8/9/32 | 56 | 20 | 32 | 41 | 24.485 | 0.208 |
| L lateral frontal cortex | 8 | 62 | -19 | 23 | 50 | 22.796 | 0.197 |
| R superior frontal gyrus | 10 | 58 | 23 | 56 | 17 | 19.641 | 0.174 |
| L superior temporal gyrus/superior parietal lobule | 39/7 | 161 | -31 | -55 | 29 | 27.979 | 0.231 |
| R precentral gyrus | 6 | 39 | 44 | -14 | 44 | 29.831 | 0.243 |
| R caudate | -- | 30 | 17 | 17 | 23 | 25.346 | 0.214 |
| L vmPFC*** | 32/10 | 50 | -7 | 50 | 2 | 15.593 | 0.144 |
|  |  |  |  |  |  |  |  |
|  |  |  |  |  |  |  |  |

Note: Coordinates based on the Tournoux & Talairach standard brain template, BA= Brodmann’s Area, *** vmPFC results recorded at initial threshold of *p* < 0.005.

Table S9. Regions showing ICU-by-Feedback interactions from the ANCOVA analysis conducted on the whole sample.

| **REGION** | **BA** | **Voxels** | **X** | **Y** | **Z** | ***F*-value** | ***η*²** |
| --- | --- | --- | --- | --- | --- | --- | --- |
|  |  |  |  |  |  |  |  |
| ***ICU-by-Feedback*** |  |  |  |  |  |  |  |
|  |  |  |  |  |  |  |  |
| R rmFC | 9/32 | 32 | 8 | 38 | 29 | 21.344 | 0.116 |
| L lateral frontal cortex | 8 | 15 | -22 | 14 | 44 | 16.315 | 0.091 |
| L lateral frontal cortex | 8 | 11 | -19 | 26 | 50 | 15.575 | 0.088 |
| L precuneus | 19 | 9 | -31 | -67 | 35 | 14.845 | 0.084 |
| R precentral gyrus | 4 | 15 | 44 | -14 | 44 | 17.750 | 0.099 |
| R caudate | -- | 18 | 14 | 11 | 23 | 23.513 | 0.127 |
| L vmPFC*** | 32/10 | 61 | -7 | 50 | 2 | 16.469 | 0.092 |
|  |  |  |  |  |  |  |  |
|  |  |  |  |  |  |  |  |

Note: Coordinates based on the Tournoux & Talairach standard brain template, BA= Brodmann’s Area, *** vmPFC results recorded at initial threshold of *p* < 0.005.

Kemp, E. C., Frick, P. J., Robertson, E. L., Ray, J. V., Thornton, L. C., Wall, T. D., . . . Cauffman, E. (2020). *Clinical cutoff scores for the Inventory of Callous-Unemotional traits (ICU) predict higher rates of juvenile delinquecy.* Paper presented at the American Psychology-Law Society New Orleans, LA.
